# High incidence of sexual dysfunction and timed intercourse was found only in infertile males who with known impairment of sperm quality for a long period: evidence from a hospital-based cross-sectional study

**DOI:** 10.1186/s12958-022-01010-4

**Published:** 2022-09-16

**Authors:** Xiaowei Yu, Songling Zhang, Linjiao Chen, Xiao Yuan Zhang, Qun Wang

**Affiliations:** 1 Department of Reproductive Medicine, Department of Prenatal Diagnosis, The First Hospital of Jilin University, Changchun, Jilin, China; 2 Department of Obstetrics and Gynecology, The First Hospital of Jilin University, Changchun, Jilin, China

**Keywords:** Male infertility, Sexual dysfunction, Sperm quality, Timed intercourse

## Abstract

**Background:**

Infertile men with higher sexual dysfunction risk and increased psychological burden, were also associated with more inclined to timed intercourse. Decreased semen quality may have adverse effects on male sexual function. However, it is also likely that many of these sequences do not play a direct role, those negative consequences may depend mainly on the later failed attempting pregnancy. Research is limited in this area.

**Methods:**

This cross-sectional study was based on a group of 509 men who were assessed for couple’s infertility at the First Hospital of Jilin University between June 2021 and October 2021. All the men completed a comprehensive questionnaire, and then were divided in two groups. Group A included patients who either never received a routine infertility work-up or done so recently within the last 6 months. Group B included patients who previously received a sperm quality assessment at least 6 months or more prior. Patients were further categorized into three subgroups according to the severity of the decreases in their sperm parameters: severe, mild-moderate, and normozoospermic.

**Results:**

The prevalence of erectile dysfunction was higher in Group B Mild-Moderate and Group B Severe in comparison to Group A (OR=1.86 [1.07–3.24], *P *= 0.027; OR=5.312 [2.69–10.49], *P *< 0.001, respectively). No significant differences were found between Group A and Group B-normozoospermic. Similar results were observed in the prevalence of premature ejaculation between the groups. Timed intercourse was seen in 11.8% (20/170) of men in Group A and 16.2% (19/117) in Group B-normozoospermic. It was more commonly practiced among infertile men in Group B-Mild-Moderate and Group B Severe, as 28.2% (44/156) and 25.7% (17/66) of these couples had attempted to conceive through timed intercourse (*P *< 0.001).

**Conclusions:**

Our findings indicate that the severity of sperm quality impairment was negatively associated with sexual dysfunction only in infertile men who with known impairment of sperm quality for a long period. Timed intercourse was more common among these couples. For those individuals had never test their sperm quality, although more than half of these patients showed a decrease in sperm quality, the incidence of sexual dysfunction is relatively low and were comparable to those men examined previously known as normozoospermic.

## Introduction

Sexual dysfunction (SD) is highly prevalent in male partners of infertile couples [1]. Sperm defects includes decreased sperm count, reduced sperm motility and abnormal morphology, which may not be the direct cause of male SD, as studies on the subject have revealed that SD in infertile males is possibly associated with psychological disorders [1–3]. Still, few studies have focused on sperm quality disturbances in SD among infertile male patients.

The need for a second birth rapid surge since the family planning policy changed in China, male SD prevalence and consequent burden are expected to rapidly increase [4]. Studies of the relationship between SD and sperm quality are scarce [5–11], since most studies so far have focused on azoospermia [5–9, 11]. Learning of their azoospermia diagnosis can result in severe mental trauma for a couple trying for pregnancy, as the capacity to natural conception has been considered either extremely rare or non-existent, feelings of frustration and fears of being judged or labeled, as a result, patients with azoospermia were more likely to experience impotency [5]. A much higher incidence of SD was also observed in azoospermia patients when compared with fertile population [6]. Advances in intracytoplasmic sperm injection technology have allowed for the use of spermatozoa obtained by epididymis or testicular sperm extraction, making it possible for many azoospermia men to become biological fathers [7]. This has created a sense of hope for these men who might otherwise have been unable to have a natural pregnancy [8]. Still, despite these advancements, an unsuccessful testicular sperm extraction procedure can result in the intensification of SD in azoospermia men [9].

Adequate numbers of normal sperms and engaging in intercourse around the time of ovulation are the two most important factors in achieving a natural pregnancy. However, when couples resort to planned intercourse, sexual activity is restricted to conception rather than recreation. Some infertile couples choose to allocate more of sex lives to the time period of fertile window in order to maximize their chances of conception, which is referred to as timed intercourse (TI). However, this kind of obligatory intercourse separates sex from sexuality and can result in situational erectile dysfunction (ED) [12, 13]. TI failure results in the most prevalent form of situational ED, in which the males only experience ED during their wives’ ovulatory time [14]. This dramatically increases the physical and psychosocial burdens for the couple [14, 15]. Previous studies have revealed that male sexual functions show a downward trend in relation to low sperm concentration [10]. However, evidence for the relationship between TI and sperm quality was absent, so no cases were further investigated.

Our first working hypothesis is that the SD in infertile males is associated with decreased sperm quality, but do not immediately contribute to male SD and those negative consequences may depend mainly on the later failed attempting pregnancy, which also cause psychological problems (eg, anxiety) among male partners of infertile couples. Thus, our primary objective was to investigate the possible association between sperm quality and SD, here infertile males were classified into 2 groups: Patients who had never test either or had only recently routine infertility work-up; Patients who previously received a sperm quality assessment 6 months or more prior.

Our secondary hypothesis was that those male partners of infertile couple who previously received a sperm quality assessment with a known decreased sperm quality, choose to allocate more of sex during their wives’ ovulatory time. Thus, our secondary objective was to investigate the relationship between TI and sperm quality in infertile couples.

## Patients and methods

This study was a cross-sectional cohort study on a group of 509 men that were assessed at a single Reproductive Center at the First Hospital of Jilin University between June 2021 and October 2021 (ClinicalTrials.gov identifier number: NCT04941690). The study was approved by the First Hospital of Jilin University ethics committee (21K064-001) and participants signed informed consent. Male patients enrolled were: i, greater than or equal to 20 years old and seeking medical care for couple infertility. ii, Men living together with wives and have regular intercourse during the study period; iii, Couples planned to try to conceive; iv, Men voluntarily came to the andrology clinic to seek medical help. The exclusion criteria were: i, Men with a previous physician diagnosis of severe cardiovascular diseases, hypogonadism, and brain strokes; ii, Men separated with their wives or sexual intercourse ≦1 time per month (such as during in vitro fertilization treatment, female surgical treatment or vaginal operation prohibits sexual intercourse); iii, Men who with psychopathological conditions or were receiving medications that may affect sexual function (such as phosphodiesterase 5 inhibitors, testosterone, and selective serotonin reuptake inhibitors).

TI is considered that more than 70% of sexual intercourse in a month is concentrated around the time of ovulation. The predicted ovulation day using various ovulation prediction methods such as calendar charting, tracking basal body temperature, cervical secretion investigation, and urinary hormone measurement. Infertility is defined as the inability of a sexually active couple to achieve pregnancy within one year of engaging in unprotected intercourse [16].

All participants completed a web-based questionnaire that included comprehensive demographic information, as well as the five-item version of the International Index of Erectile Function (IIEF-5) for diagnosis of ED, the Intravaginal Ejaculatory Latency Time (IELT) and Premature Ejaculation Diagnostic Tool (PEDT) for diagnosis of premature ejaculation (PE). The IELT was measured from the initiation of vaginal penetration until ejaculation in the most recent attempt at sexual intercourse, was determined by the self-statement of the male participants. To assess whether the association between the presence of awareness of sperm alterations and SD was partly mediated by anxiety, anxiety was assessed by the seven-item Generalized Anxiety Disorder (GAD-7) Scale.

Two groups were divided according to the infertile couple’s knowledge of their sperm quality; Group A included patients who had either recently routine infertility work-up within the last six months or had never done so at all. Group B included patients who previously received a sperm quality assessment6 months or more prior, were also retested in our outpatient center. We chose to set six months as our time point since questions in IIEF-5 and PEDT ask about the last 6 months sexually experiences before the start of the investigation. Since most of the male attended the clinic on his own, we were unable to evaluate the fertility and sexual function in females.

To assess if sperm characteristics were associated with male SD, semen samples were collected in sterile containers through masturbation after three to seven days of sexual abstinence. According to the parameters on the severity of decline in sperm progressive motility, concentration, and morphology established by the WHO, patients were categorized into three subgroups. The severe group included men who were oligozoospermic with a concentration <5 × 10^6^ sperm/mL, or/and asthenozoospermic with a progressive motility of <10%, or/and teratozoospermic with <1% normal morphology. Mild to moderate group included oligozoospermic men whose concentrations ranged between 5 to <15 × 10^6^ sperm/mL, or/and asthenozoospermic men who displayed progressive motility ranging between 10% to <32%, or/and men with normal morphology falling between 1 to <4. The normozoospermic group included those with ≥15 × 10^6^ sperm/mL, ≥32% progressive motility, and ≥4% normal morphology.

The statistical significance of differences in mean and proportion was evaluated with a one-way analysis of variance and the Pearson chi-square test for comparisons of more than two groups. Unpaired two-sided Student’s t-tests or Mann-Whitney U-test were used for comparisons of the two groups. All primary and secondary variables were first examined using an exploratory data analysis method and recorded descriptively. Variables were retained for analysis when deemed clinically significant to the results. Subsequent multivariate analyses adjusted for potential confounders and binary logistic regression analyses were performed for categorical parameters, while analysis of covariance (ANCOVA) was used for continuous parameters. The odds ratios (ORs) and 95 % confidence intervals (CIs) were calculated. *P*-values < 0.05 were considered statistically significant.

## Results

### Participants

Among the 509 males of the infertile couples, the 170 individuals in Group A were recently or had never been tested, and thus were unaware of their sperm quality or had only been aware for a short time. The 339 participants in Group B had completed a sperm quality assessment at least six months prior, and thus had been aware of their sperm quality for some time. 117 members of Group B were normozoospermic (Group B-N), while other the 222 men with at least one abnormality in their sperm parameters were divided into two groups depending on the degree of disease severity. 156 patients were Mild-moderate (Group B-M) and 66 patients had the Severe form (Group B-S).

In Group A we found 4.2% (7/117) subjects had azoospermia, and 4.4% (15/339) of individuals in Group B had the same affliction. In the group overall, 19.1% (97/509) of subjects showed isolated abnormalities in sperm parameters, including oligozoospermia, asthenozoospermia or teratozoospermia. 37.8% (192/509) had at least two abnormalities in their sperm parameters. Table 1 reports the clinical characteristics of the participants. There were significant differences in their ages, monthly household incomes, frequency of night shifts, duration of infertility, and engagement in TI that was observed between Group A and Group B (Table 1).

**Table 1:** Demographics, clinical and seminal parameters of the subjects studied

**Demographics and clinical parameters**	**Group A (170)**	**Group B (339)**	***P*** ** value**
Age (years)	31.9 ± 4.35	32.9 ± 4.39	**0.011**
BMI (kg/m^2^)	25.34 ± 3.82	26.22 ± 3.78	0.914
Current smokers (%)	50.6	46.9	0.433
Current alcohol consumption (≥4 drinks/week) (%)	19.4	15.6	0.283
Education level (%)			0.478
No higher than high school	21.2	20.1	
High school	24.1	29.2	
University and above	54.7	50.7	
Monthly household income			**0.039**
5000	32.9	32.7	
5000-7000	54.1	45.4	
7000~	12.9	21.8	
Night shifts (times/week) (%)			**<0.01**
1-2	34.1	13.6	
3-4	7.1	5.3	
>4	0.6	0.9	
Type of infertility			0.652
Primary	95.9	95	
Secondary	4.1	5	
Duration of infertility (years) (%)			**<0.01**
≤ 2	62.9	38.1	
2-4	25.9	31.9	
>4	11.2	30.1	
Timed intercourse (%)	11.8	23.6	**<0.01**
Mean testis volume (Prader) (ml)	13.1 ± 3.18	13.2 ± 3.30	0.590
Clinical varicocele (Palpation) (%)	10.0	11.8	0.544
Symptoms of Prostatitis (%)	5.3	7.7	0.318
Seminal parameters			
Sexual abstinence (days)	4.13 ± 1.48	4.14 ± 1.44	0.893
Semen volume (ml)	3.61 ± 1.41	3.62 ± 1.54	0.184
Sperm concentration, ×10^6^/ml	65.68 ± 51.94	54.42 ± 45.15	0.472
Sperm progressive motility (%)	33.89 ± 18.09	29.10 ± 17.29	0.735
Sperm normal morphology (%)	4.03 ± 2.05	3.73 ± 2.04	0.399

Data are expressed as mean ± SD when normally distributed, as medians (quartiles) for parameters with non-normal distribution, and as percentages when categorical. Bold characters emphasize significant associations.

Potential importance in univariate analyses on aforementioned clinical characteristics were included in further analyses as potential confounders.

### Sexual function

**Fig. 1:**
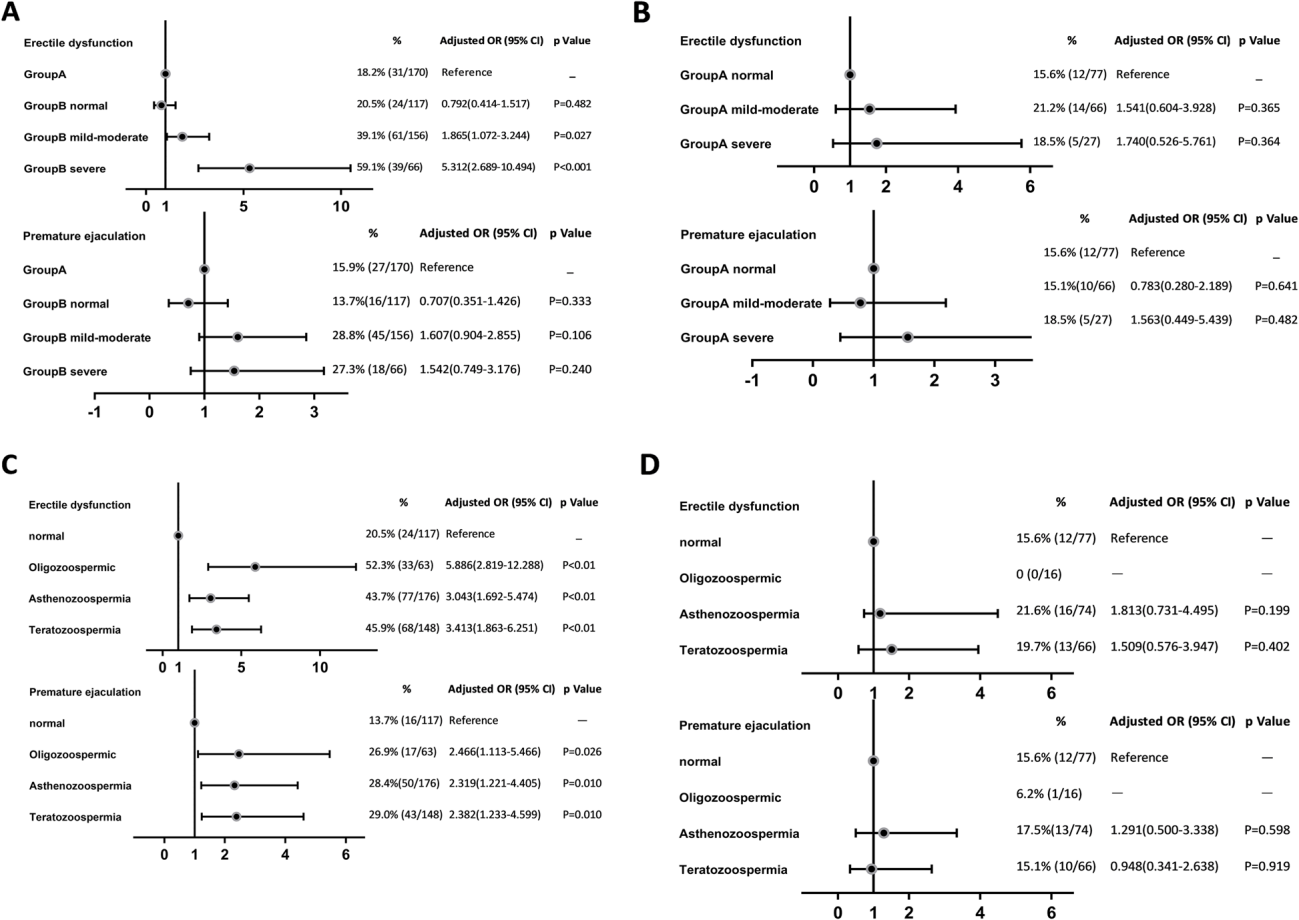
Comparison of sexual dysfunction according to impairment of sperm quality among the groups. **A** Comparison prevalence of sexual function among patients in Group B with the severity of sperm quality impairment, with Group A as reference. **B** Comparison prevalence of sexual function among patients in Group A with the severity of sperm quality impairment; **C**, **D** Comparison prevalence of sexual function among patients in Group B (**C**) and Group A (**D**) with the type of sperm quality impairment, with normozoospermic men as reference. Potential importance in univariate analyses on the aforementioned clinical characteristics were included in analyses as potential confounders (age, the participant’s monthly household income, frequency of night shifts, duration of infertility, and engagement in TI). Groups A indicate: Patients who either never received a routine infertility work-up or done so recently within the last 6 months; Groups B indicate: Patients who previously received a sperm quality assessment at least 6 months or more prior

IIEF-5 scores were available from all our participants. In our population, 30.6% (156/509) reported ED (with an IIEF-5 score ≤ 21). Of these patients, 67.9% (106/156) exhibited mild to moderate ED symptoms (IIEF-5 score 12 to 16), while 5.3% (18/339) had severe ED (with an IIEF-5 score ≤ 11) in Group B only. Indeed, none of the subjects in Group A had severe ED. After adjusting for potential importance in univariate analyses on age, the participant’s monthly household income, frequency of night shifts, duration of infertility, and engagement in TI, the number of infertile males who developed any kind of ED was higher in Group B-M and Group B-S when compared with Group A (OR=1.86 [1.07–3.24], *P* = 0.027; OR=5.312 [2.69–10.49], *P *< 0.001; respectively). No significant differences were found between Group A and Group B-N. Similar results were observed in the prevalence of PE as seen in Fig. 1A.

Figure 1C compares the difference in prevalence of SD between men with normozoospermic and abnormal sperm parameters including oligozoospermia, asthenozoospermia or teratozoospermic in Group B. After adjusting for potential importance in univariate analyses on age, the participant’s monthly household income, frequency of night shifts, duration of infertility, and engagement in TI , the results showed an increase prevalence of SD in men with oligozoospermia, asthenozoospermia or teratozoospermia, the highest OR was identified in oligozoospermic group (OR = 5.89 [2.82–12.29], *P *< 0.001).

Next, we further investigated whether the impaired sperm quality trends corresponded with a higher prevalence of SD in Group A. After adjusting for potential importance in univariate analysis, SD rates were separately compared in terms of degree of decline and types with the normozoospermic group. We did not find significant evidence of association between impaired sperm quality and SD in Group A, as seen in Fig. 1B and D.

### Anxiety and Timed intercourse

**Fig. 2:**
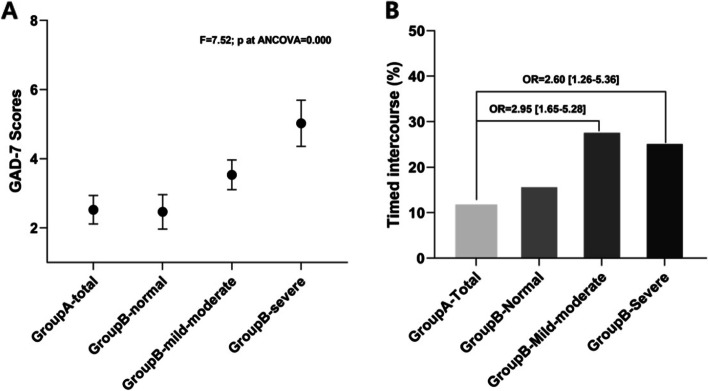
Comparison among groups for timed intercourse and GAD-7 Score. Groups **A** indicate: Patients who either never received a routine infertility work-up or done so recently within the last 6 months; Groups **B** indicate: Patients who previously received a sperm quality assessment at least 6 months or more prior. The insets show the age-adjusted comparison among groups

The more decreased in spermatozoa quality, the higher total GAD-7 scores were observed which indicate more severe anxiety symptoms. Unsurprisingly, the GAD-7 scores were highest in the Group B-S, followed by Group B-M, both showed higher GAD-7 scores compared to Group A and Group B-N (*P *< 0.001). No significant differences in GAD-7 scores were found between Group A and Group B-N (Fig. 2A).

TI was seen in 11.8% (20/170) of men in Group A and 16.2% (19/117) in Group B-N. It was more commonly practiced among infertile men in Group B-M and Group B-S, as 28.2% (44/156) and 25.7% (17/66) of these couples had attempted to conceive through TI (*P *< 0.001). Although men in Group B-S showed the worst sexual function, TI was comparably prevalent in these men in comparison to Group B-M (*P *= 0.186) (Fig. 2B).

## Discussion

To our knowledge, this is the first study to investigate differences in SD between infertile couples who were aware of their sperm quality for a longer period and those who recently or had never conducted a routine infertility work-up. We sought to investigate the association between the severity and type of decline in sperm quality and SD in males of infertile couples in comparison with the results of those who had been previous tested known as normozoospermic. At the same time, we also explored the relationship between TI and sperm quality in infertile couples.

We found that an increased prevalence of SD only in those men who had known of their reduced sperm quality for a longer period. In men who had recently or never received a routine infertility work-up and had thus had knowledge of their sperm quality for a shorter amount of time or not at all, the prevalence of SD did not increase with the severity of their sperm quality impairment, despite similar sperm quality of semen volume, concentration, progressive motility, and morphology in the two groups. Compared to recently or never tested subjects, those with known sperm quality as normozoospermic individuals experienced similar SD incidence rates. All males who had knowledge of their declined sperm quality for a long period exhibited an increase in SD rate that were closely associated with increased anxiety. In our study, patients who had been previous tested and were aware of impaired sperm quality hoped to maximize their chances of conception, and thus were more inclined to engage in TI.

In our study, ED prevalence was similar to previously reported rates in infertile couples in China [3, 17, 18]. ED prevalence gradually increased with the severity of sperm quality impairment among men who had previous knowledge of reduction in sperm quality, but not those who had recently or never tested. Some ED-related parameters, such as age, the participant’s household income, duration of infertility, and engagement in TI were different among the seminal groups, and this disparity remained after controlling for these factors.

The prevalence of PE observed in the present study is similar to what has been reported in the general population. In this study, the prevalence of PE observed in recently or never tested infertile men was 15.9% and 13.7% for normozoospermic men who had previous knowledge of their sperm quality, which is similar to the rate found in the general Chinese population(11.2%) [19]. Previously tested men with poor sperm quality with mild to moderate symptoms (28.8%) and severe symptoms (27.3%) also showed a higher but comparable PE frequency in our study, which is in line with a previous study [6].

Although the declined sperm quality in the recently or never tested group is comparable to the group with previous knowledge, overall SD rates were low in recently or never tested group and comparable to normozoospermic men in the group with previous knowledge. This could be due to several factors. First, there were still many males who had never been tested for sperm quality among the infertile couples, with a proportion of up to 33.4% (170/509) in our study. Although these infertile couples did not use any contraception, they did not deliberately attempt pregnancy and attributed their infertility to they themselves not wanting the child. Second, the recently or never tested group and normozoospermic men in the group with previously knowledge showed comparable anxiety score, and the anxiety in these two seminal groups may simply be due to the infertility itself. Consistently, we also found that the association between awareness of sperm alterations and SD was partly mediated by anxiety. The more decreased the spermatozoa quality, the more severe the anxiety symptoms was, the GAD-7 scores were highest in the Group B-S. This is consistent with many previous studies in the field, infertile men may develop feelings of inadequacy, guilt, depression, and distress that can be further exacerbated by knowledge of declined sperm quality [1, 20], particularly since severely decreased sperm quality causes great frustration for males experiencing infertility [8].

Human reproduction is a relatively inefficient process, with the chances of conception in a given instance of intercourse estimated to be between 0.1% and 9.7% [21]. However, sex is a basic human instincts and procreation is not the sole purpose of human sexuality. Relevant psychological and behavioral changes were indeed noted in infertile couples who were attempting pregnancy [22]. Timed intercourse targeting the period of ovulation has been hypothesized to maximize the chance of conception [23]. However, forcing obligatory sex at a particular time separates sex from sexuality and can also result in the male experiencing feelings of psychological distress and anxiety during the pregnancy attempt that lead to SD [24]. We found infertile couples were more inclined to arrange their sexual lives during the fertility window. In fact, 11.8% of recently or never tested infertile men and 16.2% of previously tested normozoospermic men frequently engaged in TI. This behavior was more pronounced in previously tested infertile men with knowledge of their mild to moderate declined in sperm quality, as 28.2% of these couples have attempted to achieve conception through TI to compensate for the declined sperm quality. Previously tested men who were aware of their severe reduction in sperm quality showed the worst sexual function and anxiety scores. Interestingly, TI was comparably prevalent in previously tested infertile men who were aware of their mild-moderate declined in sperm quality (25.7% versus 28.2%). It could be speculated that because men who experienced a severe declined in sperm quality were aware that the sexual act had low chances of producing a pregnancy, their desire to engage in intercourse was reduced and as a result they are more likely to experience both depression and anxiety [20].

This study had several limitations. First, as shown in previous study, the male sexual function declined with lower sperm count, the partner's Female Sexual Function Index scores were also significantly associated with male partner sexual function; The coexist of sexual dysfunction in the female partner could also contribute to the deterioration of erectile function [10]. Unfortunately, in conservative cultures like China, most of the male attended the clinic on his own; Also the sexuality, sexual health, and sexual functioning of women and their ability to express their attitudes and feelings toward it are still considered inappropriate. So we do not have the clinical data available. This is a major limitation of this study. In addition, regardless male or female, physical activity can effectively reduce the occurrence of SDs, this effect was partly mediated by body uneasiness, psychopathological symptoms and sexual distress [25]. It is a pity that we did not evaluate the effect of physical activity on sexual dysfunction in this study, and some relevant data (such as body uneasiness, psychopathological symptoms and sexual distress) were missing, is another limitation. Second, it is known that hormone can play an important role in male SDs, but these data were not recorded in our study, we recognize this as a significant limitation to our study. As it is widely accepted that oxytocin is actively involved in regulating orgasm and ejaculation and there are also links between the delayed, premature ejaculation and hypothyroidism, hyperthyroidism; In the meantime, anxiety could also reduce testosterone level and which then influences sexual dysfunction [26, 27]. Testing the serum testosterone level is critical during the male SD management, as in addition to the regulation sexual desire and NO release, testosterone may also involve in the regulation of the expression of phosphodiesterase type 5 (PDE5) [28]. The androgen-dependent PDE5 expression may partially explain the ineffectiveness of the phosphodiesterase 5 inhibitors (PDE5i) in a portion of patients with ED [29]. Third, Metabolic syndrome (MetS) has adversely effects on sperm quality and functionality [30]. In men with couple infertility, MetS is also associated with hypogonadism, ED, somatization and depression [31]; MetS males with ED are more likely results from a high E2 rather than from a low testosterone, are more likely to be insensitive to PDE5i [32, 33]. No information about metabolic was collected is also a limitation of the current study. Furthermore, it cannot be said for sure whether the individuals in this study accurately represent infertility in general, as many infertile couples resort to assist reproductive procedures that can overcome some difficulties affecting natural conception. Finally, the analyses were implemented cross-sectionally and were hospital-based, raising the possibility of selection bias. Further larger prospective studies are still needed to confirm our findings.

## Conclusions

Firstly, we show the presence of semen quality impairment did not immediately lead to the occurrence of male sexual dysfunction. Only those couples that were completed a sperm quality assessment at least six months prior, with impaired sperm quality showed much higher rates of sexual dysfunction. For those individuals had never test their sperm quality, although approximately 55% of these patients showed a decrease in sperm quality, the incidence of sexual dysfunction is relatively low and were comparable to those men examined previously known as normozoospermic. secondly, Infertile couples with previously known as declined sperm quality were more inclined to arrange their sexual lives during their fertile window and would hope maximize their chances of conception by using timed intercourse, and we did not find a similar trend in couples who had never conducted a routine infertility work-up or these normozoospermic men .Therefore, in infertile men with declined sperm quality, not only the infertile but also the sexual health of the patients should be focused on, merits dedicated investigation in future studies to improve not only reproductive but also sexual health.

## Data Availability

The datasets used and/or analyzed during the current study are available from the corresponding author on reasonable request.

## Abbreviations

SD: Sexual dysfunction
TI: timed intercourse
ED: Erectile dysfunction
IIEF-5: International Index of Erectile Function
IELT: Intravaginal Ejaculatory Latency Time
PEDT: Premature Ejaculation Diagnostic Tool
PE: premature ejaculation
GAD-7: 7-item Generalized Anxiety Disorder Scale
WHO: World Health Organization
ORs: Odds ratios
CIs: Confidence intervals
ANCOVA: analysis of covariance
MetS: Metabolic syndrome
PDE5i: phosphodiesterase 5 inhibitors
